# Exploring cost trajectories of patients admitted to geriatric rehabilitation in the Netherlands

**DOI:** 10.1093/ageing/afag058

**Published:** 2026-03-24

**Authors:** Astrid D Preitschopf, Eline D Kroeze, Margriet C Pol, Marije S Holstege, Bianca M Buurman

**Affiliations:** Department of Medicine for Older People, Amsterdam UMC Location VUmc, Amsterdam, The Netherlands; Research, Omring, Hoorn, The Netherlands; Aging & Later Life, Amsterdam Public Health Research Institute, Amsterdam, The Netherlands; Department of Medicine for Older People, Amsterdam UMC Location VUmc, Amsterdam, The Netherlands; Aging & Later Life, Amsterdam Public Health Research Institute, Amsterdam, The Netherlands; Department Internal Medicine, Section Geriatric Medicine, Amsterdam UMC Location AMC, Amsterdam, The Netherlands; Department of Medicine for Older People, Amsterdam UMC Location VUmc, Amsterdam, The Netherlands; Aging & Later Life, Amsterdam Public Health Research Institute, Amsterdam, The Netherlands; Research Group Occupational Therapy: Technology and Participation, Faculty of Health, Centre of Expertise Urban Vitality, Amsterdam University of Applied Sciences, Amsterdam, The Netherlands; Research, Omring, Hoorn, The Netherlands; Research Group Geriatric Rehabilitation, Centre of Expertise Prevention in Health and Social Care, Faculty of Health, Sports and Social Work, Inholland University of Applied Sciences, Amsterdam, NH, The Netherlands; Research, De Zorgcirkel, Purmerend, The Netherlands; Department of Medicine for Older People, Amsterdam UMC Location VUmc, Amsterdam, The Netherlands; Aging & Later Life, Amsterdam Public Health Research Institute, Amsterdam, The Netherlands; Department Internal Medicine, Section Geriatric Medicine, Amsterdam UMC Location AMC, Amsterdam, The Netherlands

**Keywords:** cost trajectories, geriatric rehabilitation, group-based trajectory modelling, older people, health claims data

## Abstract

**Objective:**

Geriatric rehabilitation (GR) is an essential component of transitional care. However, insight into longitudinal care use and related costs is limited. This study mapped GR trajectories over a six-month period, identified latent groups with similar cost patterns and examined patient-level cost drivers.

**Method:**

We analysed administrative claims data from Statistics Netherlands, focusing on total trajectory costs for GR patients admitted between 1 February and 31 July 2022. Costs included hospital care (inpatient and outpatient), district nursing, long-term care (at home and in nursing homes) and short-term residential care. We used descriptive statistics, group-based trajectory modelling and logistic regression to examine differences between the identified cost groups.

**Results:**

Over six months, 27 462 patients received GR, with a mean total cost of €40 469 (SD = €23 033). Sixty-eight percent of patients returned home, of whom 17% received home-based GR. Two latent groups were identified: a lower-cost group (88%) with mean costs of €35 024 (SD = 16 408), and a higher-cost group (12%) with mean costs of €82 012 (SD = 23 928). Patient characteristics only partially explained group membership. The higher-cost group accounted for 23% of total costs, mainly due to hospital (re)admissions, nursing home admissions and longer GR stays.

**Conclusion:**

GR costs were unevenly distributed: 12% of patients incurred 23% of total costs. Key factors associated with high costs were hospital care, nursing home admissions and prolonged GR stays. Early identification and better care coordination, including shifting care closer to home, may improve continuity, reduce costs and support more efficient geriatric rehabilitation delivery.

## Key Points

- Costs show three phases: pre-admission hospital care, geriatric rehabilitation (GR) admission and postdischarge hospital and long-term care.
- High-cost patients had longer GR stays, frequent readmissions and more nursing home use.
- Costs were skewed, where 12% of patients accounted for nearly a quarter of total costs.
- Shifting hospital care home and alignment with district nursing may reduce readmissions and improve cost efficiency.
- Future research should assess preventive disease management programmes and cost-effectiveness of home-based interventions.

## Introduction

Geriatric rehabilitation (GR) is a multidisciplinary approach aimed at restoring function in older adults who have experienced a sudden decline in functional ability due to illness or an acute event [1]. After being discharged from hospital, patients who are not immediately able to return home often receive GR in a skilled nursing facility, with rehabilitation continuing at home when feasible, referred to as home-based GR [2]. GR is one of three intermediate care models provided in the Netherlands. The others are short-term residential care (STRC) and the acute geriatric community hospital. Appendix 1 offers a description of these models.

In the Netherlands, GR is embedded within a broader system of acute and postacute care—including hospital care, district nursing, long-term care (LTC) and STRC—regulated by three distinct healthcare acts governing services for older adults. GR patients receive support from multiple professionals across various organisations and funding systems before, during and after rehabilitation. Policy should therefore promote coordination between these services rather than treating them as separate entities. This is essential to ensure appropriate care at the right time and place, supporting recovery of independent functioning [3]. Changes in one part of the care continuum inevitably impact others. For example, the trend to shorten hospital stays can increase the complexity of care in GR [4, 5]. Simultaneously, national policies promoting ageing in place align with the preferences of older adults but frequently lead to patients entering GR at more advanced stages of frailty. These trends intensify pressure across the care pathway, underscoring the need for ongoing innovation in service delivery across various settings, particularly in light of demographic ageing and workforce shortages [2, 3].

To address these challenges, various initiatives have concentrated on transitioning from inpatient to home-based rehabilitation models [6]. Evidence supports the efficacy of both conventional and home-based GR [1, 7–10], demonstrating that home-based GR is at least as effective as institutional care in facilitating functional recovery and minimising hospital length of stay [11, 12]. Nevertheless, our understanding of how GR relates to and interacts with other sectors in the healthcare system remains limited. While national datasets provide information on patient numbers, diagnoses and length of stay (LOS) [13], the comprehensive trajectory of care from hospital admission through rehabilitation and return home is insufficiently understood.

This lack of comprehensive insight also extends to the economic dimension of GR. Although aggregated cost data are available [13], little is known about how costs evolve throughout the longitudinal rehabilitation trajectory. Understanding these cost trajectories is essential for designing financially sustainable GR services that align with shifting demographics and care preferences. Moreover, such insights can inform policymakers in developing reimbursement models and resource allocation strategies that support innovation in rehabilitation care.

This study aims to explore cost trajectories among patients admitted to GR in the Netherlands by analysing healthcare utilisation and associated cost drivers over a 6-month period, starting 1 month before GR admission and 5 months following. By identifying and characterising distinct cost trajectories, the study seeks to understand variations in healthcare use and costs before, during and after GR to inform innovative transitional care interventions.

## Methods

### Study design

This study employed a retrospective cohort design using administrative claims data to analyse longitudinal care trajectories and associated costs among GR patients in the Netherlands. The 6-month observation window captures both pre-admission care and the recovery phase. This timeframe aligns with national and international standards, as demonstrated in previous Dutch studies on postacute care costs [14] and international intermediate care research [15, 16], where follow-up periods of 1–6 months are common. Latent cost trajectory groups with similar cost patterns were identified through group-based trajectory modelling, following Nagin and Odger’s guidelines [17, 18]. For reporting, the Strengthening the Reporting of Observational studies in Epidemiology (STROBE) [19] and The REportingof studies Conducted using Observational Routinely-collected health Data (RECORD) [20] checklists were applied (Appendix 2).

### Data and study population

We reviewed claims data from Statistics Netherlands to evaluate total trajectory care costs (TTCs) for older adults admitted to GR in the Netherlands and gathered patient characteristics (Appendix 3). To minimise the impact of COVID-19 on claims data from 2021 and address incomplete availability in 2023, we focused on records of patients admitted to GR between 1 February 2022 and 31 July 2022. Claims data and patient characteristics were linked using an anonymised population register number, achieving over 99% matching. General practitioner services and social support claims were excluded due to incomplete availability. Ethical approval was unnecessary since the data were anonymised, accessible only through a secure portal and aggregated at the population level.

### Primary outcome: trajectory costs

Costs were divided into seven categories: GR (inpatient and home-based), district nursing care, LTC (at home and in nursing homes), STRC, hospital admissions and outpatient services. Costs were linked to specific care dates or evenly distributed across periods for services spanning multiple days. Daily costs were then aggregated at the monthly level to identify trends. All costs were inflation-adjusted using the national Consumer Price Index (February 2025, Statistics Netherlands) [21] and calculated from the payer perspective based on reimbursed claims data.

Total trajectory costs were calculated. Additionally, the average cost per survival day was computed to interpret the impact of mortality within the study period.

### Statistical analysis

To understand the diverse longitudinal patterns among patients, we used descriptive statistics, group-based trajectory modelling and logistic regression to identify key differences among the identified latent groups.

#### Descriptive statistics

Descriptive statistics were used to explore three aspects of the longitudinal patterns in the trajectory costs of GR: (i) average TTC over 6 months, presented as cumulative and average monthly costs across various care categories; (ii) cost patterns by diagnostic group including stroke, trauma, elective procedures (e.g. hip and knee replacements), amputation, oncology, cardiology, respiratory conditions, organ failure and other diagnoses; and (iii) the prevalence, diagnoses and costs associated with home-based GR.

#### Group-based trajectory modelling

Group-based trajectory modelling was used to identify latent subgroups based on distinct cost trajectories over a 6-month period, applying a censored normal model for continuous cost data. Models with two to six groups were evaluated, and the optimal number of groups was selected using the Bayesian Information Criterion (BIC) [22] and the Akaike Information Criterion (AIC) [23]. To determine appropriate trajectory shapes, growth terms were varied and evaluated using the BIC and AIC. Model adequacy was evaluated based on four criteria: (i) average posterior probability >0.7; (ii) odds of correct classification >5.0; (iii) alignment between estimated and actual group membership; and (iv) narrow confidence intervals around group membership probabilities [17, 24]. No formal thresholds existed for the last two criteria.

Furthermore, the identified cost trajectory groups were described using patient- and trajectory-related variables. Patient characteristics included sex, age, migration background, living situation, income, medication use and presence of dementia. Trajectory-related factors comprised costs, LOS, survival and inflow to and outflow from GR.

#### Logistic regression

Logistic regression analysis was conducted to identify patient-level characteristics associated with membership in the high-cost group. Following recommendations by Dziak *et al*., a backwards elimination procedure was applied to select the most optimal model based on the lowest BIC [25].

## Results

### Sample characteristics

Table 1 presents the patient and trajectory characteristics. During the 6-month study period, 27 462 patients received GR and were included in the cohort (see Appendix 4, Flowchart). The mean TTC per patient was €40 469 (SD = 23 033). 40% were male (*n* = 11 100), and more than half lived alone before admission (*n* = 15 714; 57%). Following GR, 18 552 (68%) patients were discharged home; among these, 40% received district nursing care. Within 6 months, 23% of the patients were readmitted to the hospital, and nearly 13% died.

**Table 1:** Descriptive statistics for the total cohort and latent cost trajectory groups

Patient characteristics	A) Total cohort *N* = 27 462	B) High-cost group *N* = 3182	C) Low-cost group *N* = 24 280
Male, *N* (%)	11 100 (40.4%)	1706 (53.6%)	9394 (38.7%)
Age, mean (SD)	78 (9.7)	74 (10.5)	79 (9.4)
Migration background	2263 (8.2%)	368 (11.6%)	1895 (7.8%)
Living situation before GR admission, *N* (%)			
Living alone	15 714 (57.2%)	1451 (45.6%)	14 263 (58.7%)
Living together	11 121 (40.5%)	1580 (49.7%)	9541 (39.3%)
Living in institution	527 (1.9%)	132 (4.2%)	395 (1.6%)
Missing	100 (0.4%)	19 (0.6%)	81 (0.3%)
Income of household in 2021, *N* (%)^a^			
Low income	8632 (31.4%)	859 (27.0%)	7773 (32.0%)
Middle income	7396 (26.9%)	765 (24.0%)	6631 (27.4%)
High income	11 129 (40.5%)	1475 (46.4%)	9654 (39.8%)
Missing	305 (1.1%)	83 (2.6%)	222 (0.9%)
Medication use in 2021, *N* (%)^b^			
0	918 (3.3%)	170 (5.3%)	748 (3.1%)
1–4	4992 (18.2%)	567 (17.8%)	4425 (18.2%)
5–9	10 428 (38.0%)	1042 (32.8%)	9386 (38.7%)
10–14	7464 (27.2%)	854 (26.8%)	6610 (27.2%)
15–19	2847 (10.4%)	407 (12.8%)	2440 (10.1%)
>20	813 (3.0%)	142 (4.5%)	671 (2.8%)
*n* ≥ 1 psychotropic drug use in 2021, *N* (%)^c^	5560 (20.3%)	624 (19.6%)	4936 (20.3%)
Dementia	3712 (13.5%)	356 (11.2%)	3356 (13.8%)
Trajectory characteristics	A) Total cohort *N* = 27 462	B) High-cost group *N* = 3182	C) Low-cost group *N* = 24 280
Trajectory costs			
Mean trajectory costs (SD)	€40 469 (€23 033)	€82 012 (€23 928)	€35 024 (€16 408)
Median trajectory costs	€34 513	€77 536	€31 616
LOS first admission GR			
Mean LOS (SD)	36.3 (26.5)	46.2 (33.6)	35.0 (25.2)
Median	30	37	30
Readmissions (after GR discharge)			
≥1 GR readmission(s)	3057 (11.1%)	780 (25.5%)	2402 (9.8%)
≥1 ED readmission(s)	5460 (19.9%)	987 (31.0%)	4473 (18.4%)
≥1 hospital admission day(s)	6425 (23.4%)	1509 (47.4%)	4916 (20.3%)
Inflow			
Home without formal care	2202 (8.0%)	322 (10.1%)	1880 (7.7%)
Home with household help	1215 (4.4%)	97 (3.1%)	1118 (4.6%)
Home with district care	935 (3.4%)	28 (0.9%)	907 (3.7%)
Long-term care at home	106 (0.4%)	8 (0.3%)	98 (0.4%)
Nursing home long-term care	260 (1.0%)	116 (3.7%)	144 (0.6%)
Short-term residential stay	639 (2.3%)	22 (0.7%)	617 (2.5%)
Hospital admission & emergency department	22 105 (80.5%)	2589 (81.5%)	19 516 (80.4%)
Outflow			
Home without formal care	5016 (18.3%)	443 (13.9%)	4573 (18.8%)
Home with household help	1838 (6.7%)	97 (3.1%)	1741 (7.2%)
Home with district care	11 086 (40.4%)	770 (24.2%)	10 316 (42.5%)
Long-term care at home	612 (2.2%)	49 (1.5%)	563 (2.3%)
Nursing home long-term care	3584 (13.1%)	650 (20.4%)	2934 (12.1%)
Short-term residential stay	458 (1.7%)	86 (2.7%)	372 (1.5%)
Geriatric rehabilitation	148 (0.5%)	19 (0.6%)	129 (0.5%)
Hospital admission	1880 (6.9%)	714 (22.4%)	1166 (4.8%)
Hospital emergency department	311 (1.1%)	37 (1.2%)	274 (1.1%)
Hospital admission & emergency department	891 (3.2%)	229 (7.2%)	662 (2.7%)
Home-based GR *n* (%)	3067 (11.0%)	505 (15.9%)	2562 (3.3%)
Survival			
Death during GR admission	836 (3.0%)	29 (0.9%)	807 (3.3%)
Death during 6-month trajectory	3425 (12.5%)	413 (13.0%)	3012 (12.4%)
Survival days^d^, mean (SD)	171 (33.9)	173 (27.1)	170 (34.7)
Costs per survival day (SD)	€252 (€162)	€494 (€212)	€220 (€123)

SD, standard deviation; GR, geriatric rehabilitation; ED, emergency department; LTC, long-term care; LOS, length of stay; STRC, short-term residential care.

^a^Low income is up to 140% of social minimum income, middle income is 140%–200% and high income is >200% of social minimum income.

^b^Based on the ATC-code system, does not include medication provided in hospitals or under the LTC act in nursing homes.

^c^Based on Anatomic Therapeutic Chemical Codes N05A (antipsychotics), N05B (anxiolytics), N05CD (benzodiazepine), N06A (antidepressants) and N06C (antidepressants in combination with psycholeptics).

^d^Survival days calculated over a 6-month (182.5 days) follow-up period.

### Longitudinal patterns in trajectory costs

#### Average trajectory costs

**Figure 1:**
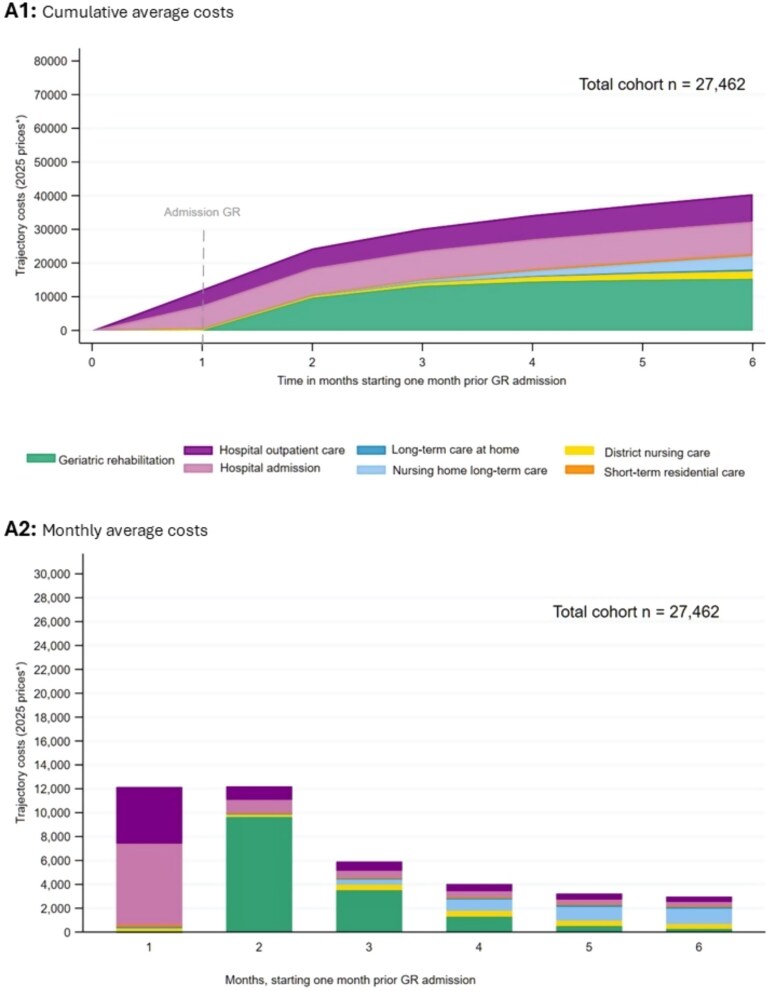
Average costs, total cohort, cumulative (A1) and monthly (A2). Costs were adjusted for inflation and are presented in February 2025 prices.

Table 1 and Fig. 1 show the average cumulative (1.A1) and monthly costs (1.A2) over the GR trajectory. Appendix 6 (Table A6.1) presents further details on the average longitudinal pattern. The GR trajectory can be divided into three phases. In the pre-admission phase (month 1), costs were mainly driven by hospital admissions (56%) and outpatient care (39%). During the GR phase (months 2–3), costs increased due to GR services and continued hospital care. In the post-GR phase (months 3–6), cumulative costs rose primarily due to nursing home LTC, which was used by 17% of patients and accounted for 10% of total trajectory costs. In month six, hospital outpatient care was still used by 49% of patients and accounted for 15% of that month’s costs. District care (6%), STRC (2%) and LTC at home (1%) contributed modestly to the TTC.

#### Diagnostic groups

Table 2 shows patient and trajectory details by diagnostic group. Trauma was most common (*n* = 8038; 29%). LOS ranged from 20 days for elective procedures to 41 days for amputations. The amputation group had the highest mean TTC (€57 417; SD = €24 897), while the elective group had the lowest (€27 565; SD = €16 321). The oncology group had the highest 6-month mortality (34.5%) and the highest mean cost per survival day (€368; SD = €214), showing high care use relative to survival time. Hospital readmission rates were highest in the amputation and oncology groups, at 38% and 39%, respectively.

**Table 2:** Descriptive statistics for diagnostic groups

Diagnoses *n* (%)	Stroke 4393 (16.0)	Trauma 8038 (29.3)	Elective surgery 3258 (11.9)	Amputation 673 (2.5)	Oncological condition 1019 (3.7)	Cardiovascular condition 1060 (3.9)	Respiratory condition 2992 (10.9)	Organ failure 3176 (11.6)	Other 2853 (10.4)	Total 27 462 (100)
Patient characteristics										
Age mean (SD)	78 (9.0)	81 (9.3)	78 (8.4)	73 (10.0)	73 (9.7)	80 (9.1)	77 (10.1)	78 (10.4)	77 (10.0)	78 (9.7)
Male *n* (%)	2168 (49.4%)	2460 (30.6%)	775 (23.8%)	476 (70.7%)	480 (47.1%)	493 (46.5%)	1466 (49.0%)	1495 (47.1%)	1287 (45.1%)	11 100 (40.4%)
Migration *n* (%)	434 (9.9%)	494 (6.2%)	275 (8.4%)	91 (13.5%)	82 (8.1%)	107 (10.1%)	290 (9.7%)	273 (8.6%)	217 (7.6%)	2263 (8.2%)
Trajectory characteristics										
Trajectory costs										
Mean trajectory costs (SD)	€42 748 (€23 540)	€37 576 (€19 204)	€27 565 (€16 321)	€57 417 (€24 897)	€50 151 (€25 557)	€47 137 (€26 303)	€42 107 (€24 677)	€45 922 (€25 188)	€42 160 (€23 516)	€40 469 (€23 034)
Median trajectory costs	€37 251	€32 300	€22 988	€55 772	€44 818	€42 605	€35 556	€40 390)	€38 218	€34 513
GBTM										
High-cost group *n* (%)	555 (17.4%)	510 (16.0%)	93 (2.9%)	203 (6.4%)	278 (8.8%)	258 (8.1%)	418 (13.1%)	510 (16.0%)	357 (11.2%)	3.182 (12%)
Low-cost group *n* (%)	3838 (15.8%)	7528 (31.0%)	3165 (13.0%)	470 (1.9%)	741 (3.1%)	802 (3.3%)	2574 (10.6%)	2666 (11.0%)	2496 (10.3%)	24 280 (88%)
LOS first admission										
LOS mean (SD)	42.2 (30.4)	38.7 (25.3)	26.2 (20.0)	53.2 (41.0)	31.8 (23.7)	30.1 (19.6)	31.9 (21.8)	35.1 (26.1)	37.8 (28.3)	36.3 (26.5)
LOS (median)	35	34	20	43	28	27	29	30	31	30
Home-based GR										
frequency *n* (%)	717 (23.4%)	743 (24.2%)	263 (8.6%)	114 (3.7%)	128 (4.2%)	118 (3.9%)	330 (10.8%)	326 (10.6%)	328 (10.7%)	3067 (11.2%)
Readmissions (after GR discharge)										
≥1 GR readmission(s	391 (8.9%)	747(9.3%)	331(10.2%)	143(21.3%)	160(15.7%)	106(10.0%)	370(12.4%)	419(13.2%)	390(13.7%)	3057 (11.1%)
≥1 ED readmission(s)	776(17.7%)	1257(15.6%)	428(13.1%)	155(23.0%)	271(26.6%)	267(25.2%)	787(26.3%)	898(28.3%)	621(21.8%)	5460 (19.9%)
≥1 hospital admission day(s)	852 (19.4%)	1405(17.5%)	510(15.7%)	256(38.0%)	399(39.2%)	326 (30.8%)	872(29.1%)	1074(33.8%)	731 (25.6%)	6425 (23.4%)
Survival										
Death during GR admission	166(3.8%)	179(2.2%)	15(0.5%)	17(2.5%)	49(4.8%)	76(7.2%)	126(4.2%)	118(3.7%)	90(3.2%)	836 (3.0%)
Death during 6-month trajectory	423(11.9%)	733(9.1%)	79(2.4%)	81(12.0%)	352(34.5%)	244(23.0%)	494(16.5%)	575(18.1%)	344(12.1%)	3425 (12.5%)
Survival days^a^, mean (SD)	171(34.7)	174(28.6)	180(15.2)	172(31.5)	151(49.2)	160(45.6)	167(40.0)	166(38.8)	172(33.0)	171 (33.9)
Costs per survival day (SD)	€264(€157)	€225(€127)	€156(€101)	€347(€158)	€368(€214)	€325(€236)	€271(€173)	€293(€172)	€259(€160)	€252 (€162)

GBTM, group-based trajectory modelling; LOS, length of stay; GR, geriatric rehabilitation; ED, emergency department.

^a^Survival days calculated over a 6-month (182.5 days) follow-up period.

#### Home-based GR

Details of home-based GR are provided in Appendix 7, Table A7.1 During the 6 months, 3067 patients (11% of 27 642) received home-based GR, representing 17% of the 18 552 patients discharged home after GR. Among these home-based GR patients, stroke (23%) and trauma (24%) were the most common diagnoses. In 60% of cases, home-based GR lasted 7 days or less. The mean TTC of GR trajectories, including home-based rehabilitation, was €44 869 (SD = €24 926). Within this group, 27% experienced GR readmission, 36% a hospital admission and 26% an emergency department visit.

### Latent cost trajectory groups

#### Identification

**Figure 2:**
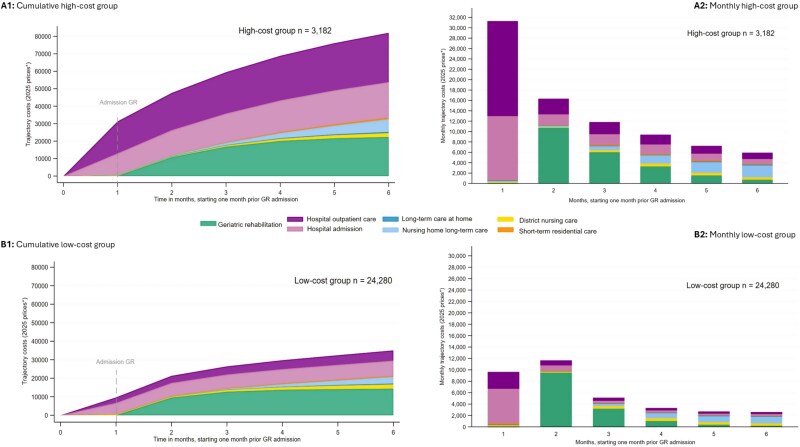
Longitudinal pattern for the two latent cost trajectory groups (cumulative and monthly cost graphs), A1 and A2 show the higher cost group, B1 and B2 the lower cost group, GR, geriatric rehabilitation; STRC, short-term residential stay; LTC, long-term care. Costs were adjusted for inflation and are presented in February 2025 prices.

Model fit statistics indicated the best fit for a censored normal model with two trajectory groups. Both groups followed a quadratic (second-order) polynomial pattern. The selected model demonstrated high classification accuracy, with average posterior probabilities of 0.93 and 0.99 and odds of correct classification of 100.88 and 10.10. Full details of the group-based trajectory modelling specifications, including model selection criteria (BIC/AIC), growth terms and diagnostics, are provided in Appendix 5 (Tables A5.1–A5.5 and Fig. A5.4). The higher-cost group (*n* = 3182) has a consistently steeply increasing cost pattern (Fig. 2.A1 and A2), with a mean TTC of €82 012 (SD = €23 928). The lower-cost group (*n* = 24 280) shows an early cost rise until month 3, followed by a more gradual increase (Fig. 2.B1 and B2), with a mean TTC of €35 024 (SD = €16 408). The higher-cost group had significantly higher hospital admissions and outpatient care costs before and after GR. Nursing home costs in this group rose from €750 in month 3 to €2291 in month 6, compared to €415 and €1148, respectively, in the lower-cost group (Appendix 6, Tables A6.2 and A6.3). In the higher-cost group, 29% of patients used nursing home LTC, accounting for 9% of their TTC (Appendix 6, Table A6.2).

#### Profile

The TTC for the GR population was €1111 billion over 6 months, based on 27 462 patients with a mean cost of €40 469 per patient. A small proportion of patients (12%, *n* = 3182) accounted for 23% of total costs, with a mean TTC of €82 012.

Table 1 shows patient and trajectory characteristics stratified by cost groups. The higher-cost group had the longest mean GR stay (46.2 days). Medication use and dementia were similar between groups. However, diagnoses such as amputation, oncological, cardiovascular, respiratory conditions and organ failure were more frequently observed in the higher-cost group.

Readmissions to GR, the emergency department and the hospital were significantly more frequent among higher-cost patients. Notably, 47.4% of this group had one or more hospital readmissions, compared to 20.3% in the lower-cost group. Nursing home LTC use was also higher (29% vs. 16%).

### Predictors of high-cost group membership

The logistic regression identified patient characteristics associated with higher healthcare costs. After backwards elimination, migration background, income and psychotropic medication use were excluded from the final model (Appendix 6, Table A6.4). In the full sample (*n* = 27 462), older age was associated with lower odds of high-cost group membership (OR = 0.96; 95% CI: 0.94–0.98). Institutional living demonstrated a strong positive association (OR = 4.51; 95% CI: 3.63–5.62), and male sex had modestly higher odds (OR = 1.36; 95% CI: 1.25–1.47).

**Figure 3:**
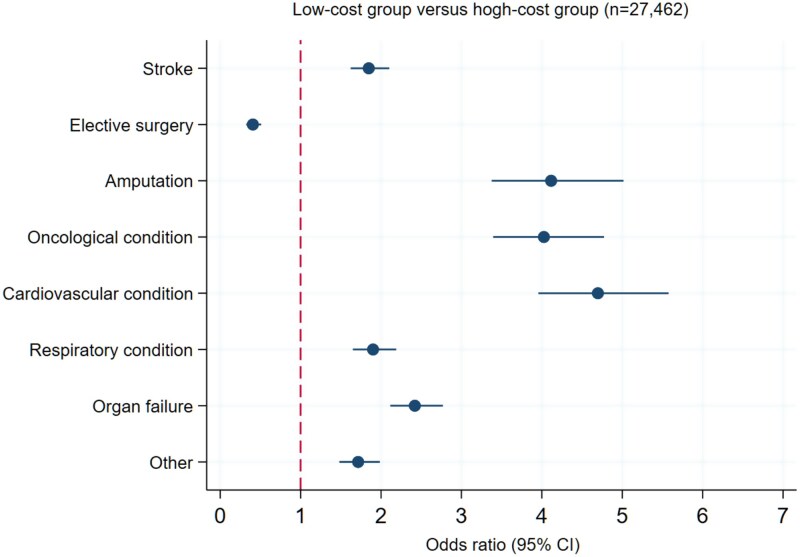
Multinominal logistic regression; log-scale forest plot being in the higher-cost group versus the lower-cost group. The reference diagnosis group, which has value *1* on the *x*-axis, where patients with trauma as the primary diagnosis of the GR admission (*P < .001).

The likelihood of high-cost group classification also varied by primary diagnosis (Fig. 3). Trauma was used as the reference category due to its large sample size (*n* = 8038), average LOS and clinical profile. Diagnoses such as cardiovascular disease, amputation and oncology were associated with over four-fold increased odds of high-cost group membership. Conversely, elective surgery was associated with significantly lower odds of being in the high-cost group (OR = 0.41; 95% CI: 0.32–0.51). The model fit was modest (McFadden pseudo-*R*^2^ = 0.101), indicating limited but non-negligible explanatory power [16].

## Discussion

This study offers new insights into the longitudinal cost trajectories of patients receiving GR in the Netherlands. By mapping healthcare utilisation and associated costs over 6 months, starting 1 month before GR admission and continuing for 5 months after, we identified three distinct cost phases: hospital care before GR, GR-related services during admission and postdischarge costs driven by ongoing hospital use and nursing home LTC. Costs varied significantly by diagnosis: trauma was the most common, while amputations incurred the highest average costs. Home-based GR, received by 17% of patients discharged home—mainly poststroke or trauma—was typically brief but linked to high readmission and acute care utilisation rates. Two cost profiles emerged, with the high-cost group showing longer GR stays, more hospital readmissions and greater nursing home usage. Patients with lifestyle-related conditions, such as amputation and cardiovascular disease, were more likely to follow this high-cost trajectory. Notably, 12% of patients accounted for 23% of total costs, underscoring substantial variation in resource intensity and care complexity.

This skewed cost distribution observed in this study aligns with previous research in intermediate and postacute care settings. In the Hospital at Home study, Ribbink *et al*. [14] found that 10% of patients were responsible for over half of the total costs, while Colón *et al*. [26] reported limited functional recovery among high-cost trajectories. These findings suggest that cost trajectories are not only financial indicators but also reflect underlying clinical complexity and recovery potential. Nonetheless, higher costs are not inherently indicative of inefficiency; they may instead reflect appropriate resource use in response to complex care needs or efforts to maintain or enhance patients’ quality of life, as previously suggested by Templeton *et al*. [27].

Three main factors associated with higher costs were hospital readmissions, prolonged GR stays and transitions to nursing homes, highlighting the need for better coordination across care settings. Transitions to nursing homes are often associated with frailty and complex recovery trajectories [14, 28, 29], underlining the importance of early identification of patients with complex care needs [30]. Improved triage at referral or admission may facilitate more personalised rehabilitation plans—tailored in duration, intensity and modality—to match individual needs better while containing costs [31].

High-cost trajectories were more common among patients with lifestyle-related diagnoses, such as amputations and cardiovascular diseases. Similar patterns are observed in Singapore [30] and Canada [31], where chronic conditions are associated with persistently high healthcare costs. These findings emphasise the importance of preventive strategies and integrated disease management—also during GR—to address modifiable risk factors and reduce readmissions. Disease management programmes focusing on timely risk stratification, patient education and self-management may reduce readmissions, long-term care use and improve outcomes. However, such approaches are not yet standard in GR and require further development. Moreover, the regression model, including diagnosis, explained only a small part of the variance in high-cost group membership (pseudo-*R*^2^ = 0.10). Given the observational design, results should be interpreted as associations rather than causal effects. This underscores that claims data, lacking key clinical constructs such as frailty and functional status, are insufficient for precise risk stratification in GR [32, 33]. Several strategies may improve care continuity and reduce readmissions. Delivering parts of hospital care at home (e.g. medication-based approach) and transitional care interventions have been shown to be cost-effective [28, 34] and may reduce hospital readmissions [34–37] and long-term care admissions [38] while improving physical functioning [39]. Further, closer alignment with district nursing—currently accessed by 66% of patients, accounting for 6% of total costs—offers potential for proactive monitoring and early intervention. Recommendations to shift care homewards and improve alignment with district nursing are supported by international and Dutch policy [40, 41] and by models such as Hospital-at-Home [38]. These approaches may improve cost-effectiveness and allow more efficient use of personnel, but the findings remain hypothesis-generating and require further research to guide implementation.

Finally, home-based GR may support transitions home and improve outcomes [12, 42, 43] but is currently used on a limited scale, often as brief, fragmented episodes. Slightly higher costs observed in these trajectories may reflect inefficiencies related to limited structural integration but may also be explained by population characteristics. Home-based GR patients may have used higher pre-admission care, had more complex needs or required longer rehabilitation periods than those with brief inpatient trajectories. Importantly, these findings reflect a period preceding recent initiatives to improve home-based GR [43]—blended use of technology, better care integration, closer district nursing alignment and hospital-at-home models—which are expected to increase efficiency and cost-effectiveness. Further development is needed to embed home-based GR more effectively within the broader rehabilitation pathway [2, 8, 43].

### Strengths and limitations

This study has several strengths. Together with the study by Kroeze *et al*. (under submission, 2025), it is among the first to explore cost trajectories in GR at this level of detail, utilising a large national claims–based dataset covering all relevant care sectors. Additionally, group-based trajectory modelling enabled the identification of distinct patient subgroups with unique cost patterns, offering more nuanced insights than analyses based solely on population averages.

Nonetheless, several limitations must be considered. First, while the dataset includes comprehensive cost and utilisation data, it lacks clinical outcomes, functional measures and patient-reported experiences. Consequently, it remains unclear whether higher costs are associated with better or worse outcomes. Second, the findings are specific to the Dutch healthcare system, which may limit generalisability to countries with different organisational and financial structures. However, international efforts toward harmonisation in GR research are ongoing [2, 44]. Third, while the 6-month observation period captures short- to medium-term trends, long-term outcomes and costs were not assessed. In addition, the inclusion of only 1 month of pre-admission data may underestimate baseline care use and affect interpretation of cost trajectories. Social support costs under the Social Support Act (WMO) were excluded due to incomplete data, potentially biasing cost comparisons between home-dwelling and institutionalised patients. Finally, healthcare utilisation in early 2022 may have been influenced by residual effects of the COVID-19 pandemic, potentially impacting service delivery and patient profiles.

Future research should explore how cost trajectories relate to clinical and patient-reported outcomes, to recognise necessary investments and potential inefficiencies. Priorities include reducing hospital-related costs, particularly those driven by readmissions, through strategies such as expanded use of district nursing care and appropriate shifting of hospital care to the home. Early identification of patients at risk of high-cost trajectories may enable more efficient, tailored rehabilitation. Further studies should explore the possibilities of GR-preventive disease management programmes and assess the cost-effectiveness of interventions such as home-based GR. Finally, cross-country comparisons may provide insight into how system design influences cost efficiency and care quality.

## Conclusion

This study offers new insights into 6-month cost trajectories of Dutch GR patients. Three phases emerged: pre-admission hospital care, GR admission and post-discharge costs from hospital use and nursing home LTC. Costs varied by diagnosis, with trauma being the most common and amputations and cardiovascular disease incurring the highest costs. Group-based trajectory modelling identified two distinct cost profiles: the high-cost group had more hospital readmissions, longer GR stays and greater nursing home use, with lifestyle-related diagnoses strongly associated with high costs. Notably, 12% of patients accounted for 23% of total costs. Findings underscore the importance of early identification and coordinated care, especially for high-cost patients. While shifting hospital care to home, integrating district nursing and home-based or digital health interventions are promising and policy-aligned. Further research is needed to confirm efficiency, cost-effectiveness and guide implementation.

## Supplementary Material

afag058_aa_25_2229_File002

## Research Data Transparency and Availability

The raw data used in this study are available through the Statistics Netherlands (CBS) remote access environment for authorised researchers (CBS microdata). Access requires approval from CBS and is subject to legal and privacy restrictions. The data management procedures and programming code used for this study are available upon reasonable request from the first author.
