# Supplementary material for: Exploring cost trajectories of patients admitted to geriatric rehabilitation in the Netherlands
Source: Age Ageing. 2026 Mar 24;55(3):afag058. doi: 10.1093/ageing/afag058 (PMC13011798; doi:10.1093/ageing/afag058)
Supplement: afag058_aa_25_2229_File002 [file afag058_aa_25_2229_file002.docx]

**Exploring cost trajectories of patients admitted to geriatric rehabilitation in the Netherlands**

Supplementary material

**Index**

[Appendix 1: Dutch context 1](#_Toc204865157)

[Appendix 2: Checklist for reporting (STROBE & RECORD) 3](#_Toc204865158)

[Appendix 3: Variables with data sources and underlying assumptions 7](#_Toc204865159)

[Appendix 4: Consort diagram 9](#_Toc204865160)

[Appendix 5: Group-based trajectory modelling specifications 10](#_Toc204865161)

[Appendix 6: Results 11](#_Toc204865162)

[Appendix 7: Home-based geriatric rehabilitation 13](#_Toc204865163)

# Appendix 1: Dutch context

**The Dutch healthcare system**

The Dutch healthcare system is broadly based on three principles: universal access to care, solidarity through mandatory medical insurance (which is provided to all), and high-quality healthcare services [1]. The system is shaped by several historical trends, changes and social conditions. The reforms in 2006 changed the role of the stakeholders and actors in the healthcare sector, while the 2015 reform was targeted towards containing costs related to long-term care [2].

Public and private insurance are merged through a universal social health insurance approach. All residents are required to purchase statutory health insurance from private insurers, who are required to accept all applicants. All insurers are mandated to operate as nonprofits. Financing is primarily public, through premiums, tax revenues, and government grants. The national government is responsible for setting health care priorities and monitoring access, quality and costs. Standard benefits include hospital, physician, home nursing, and mental health care, as well as prescription drugs. Citizens pay premiums, annual deductibles, and coinsurance or copayments on select services and drugs [3].

**Three basic health care-related acts apply to care for older people**

Three of the four basic health care-related acts that govern the healthcare system apply to care for older people in the Netherlands:

1. The Health Insurance Act (in Dutch: Zorgverzekeringswet) provides short-term medical care, such as general practitioner services, hospital care, prescription drugs, and mental healthcare [4]. This act accounts for the largest amount of the healthcare budget. Private insurance companies play a key role in implementing the act in a system based on “regulated competition” [1]. They must reimburse the standard benefits which are insured under the mandatory benefit package. The national government determines this statutory benefit package. Private entities provide short-term medical care financed through the Health Insurance Act, and profits may not be distributed to the stakeholders [3].

- Care for older people provided through the statutory benefit package includes, for example, hospital care, district nursing, temporary medical care for specific patient groups living at home and intermediate care (Geriatric Rehabilitation and Short-Term Residential Care).

1. The Long-Term Care Act (in Dutch: Wet langdurige zorg) provides long-term care for vulnerable groups such as older people with frailty, people with chronic illnesses, and people with severe mental or physical disabilities. Those requiring permanent supervision and 24-hour care are entitled to services under this act [1]. A special assessment centre (the CIZ) determines whether a person is eligible [4]. Long-term care is covered through a state-controlled mandatory insurance and administered by regional long-term care administrators at the behest of the central government. Care is provided by private, nonprofit organisations [1].

- The Long-Term Care Act applies to nursing at home or in a nursing home. Older people can obtain care through a contracted long-term care provider, but can also buy their own care at home through a personal budget.

1. The Social Support Act (in Dutch: Wet maatschappelijke ondersteuning) provides care to help people live independently at home and participate in society for as long as possible [1]. Social support services are financed through the municipal fund which is supplied to the (roughly 350) municipalities by the central government. The municipalities have a great deal of freedom in how they spend these funds in order to meet the requirement of the Social Support Act [3].

- The Social Support Act provides general support to older people such as domestic help, house adjustments and transport. They can obtain care through a contracted social care provider, but can also buy their own social care through a personal budget.

References:

1. Ministry of Public Health Welfare and Sport (2016) Healthcare in the Netherlands. Available from: https:// https://bit.ly/3uWLmBK
2. Kroneman M, Boerma W, van den Berg M, et al (2016) Netherlands: health system review. Word Health Organisaiton. Available from: https://bit.ly/3RvaMhs
3. Wammes J, Stadhouders N, Westert G. International health care system profiles, Netherlands. Commonwealth Fund. Available from: https://bit.ly/3RqAH9Z
4. Varkevisser M, Schut FT, Franken F et al (2023) Sustainability and resilience in the Dutch Health System. Partnership for Health System Sustainability and Resilience (PHSSR). Available from: https://bit.ly/47ZZ0CF

**Table A1.1** Bed-based intermediate care models in the Netherlands

| **Bed-based intermediate care for frail older adults in the Netherlands** | | |
| --- | --- | --- |
| **Geriatric revalidation** | **Short-term residential stay** | **Acute Geriatric Community Hospital** |
| *Definition:* Post-acute multidisciplinary (para)medic care for older and frail patients, including those with pre-existing functional decline or specific care needs. | *Definition:* Medical care for older adults with general health problems that do not require specialist care nor geriatric rehabilitation, but whose treatment and care needs cannot be met at home. | *Definition:* (Sub)acute specialized geriatric medical care for older patients with frailty. |
| *Goal:* To optimize functional capacities and support societal participation despite impairments, so that frail and/or older individuals can return home and live independently in the community. | *Goal:* Recovery so that older adults can return home and live independently in the community. | *Goal:* To provide medical specialist care for frail older adults in an adapted environment, so they can return home, live independently in the community and hospital (re)admissions are prevented. |
| *Admission route:*  Admission from hospital, ED, or home.  Referral by a medical specialist or ECP using a comprehensive geriatric assessment. | *Admission route:*  Admission from home, ED, or hospital.  Referral by GP or medical specialist. | *Admission route:*  Admission from ED.  Referral by a medical specialist. |
| *Admission criteria:*   1. Medical stability 2. Multidisciplinary rehabilitation needs 3. Frailty and/or multimorbidity 4. Motivation/preference to undergo rehabilitation treatment 5. A cognitive and physical status that allows participation in geriatric rehabilitation.   Targeted diagnoses: (1) stroke, (2) elective orthopedics, (3) trauma surgery (e.g., hip fractures), (4) amputations, and (5) other disorders (neurodegenerative diseases, oncological diseases, COPD, cardiac failure, internal- and multi-system failure). | *Admission criteria:*  No guidelines or targeted patient groups. | *Admission criteria (before 2023)*:   1. Older patient with an acute medical problem that requires hospitalization, such as pneumonia or exacerbation of chronic conditions such as heart failure 2. Geriatric conditions (e.g. delirium, cognitive/functional impairment, falls) 3. Hemodynamic stability 4. No complex diagnostic testing needed such as CT or MRI scans during admission 5. Return to previous living situation expected in 14 days |
| *Staffing:* A multidisciplinary team with special training in rehabilitation consisting of the elderly care physician, nurses, carers, physical therapists, psychologists, dieticians, social workers, and behavioral scientists. | *Staffing:* A multidisciplinary team consisting of the elderly care physician (or general practitioner), nurses, carers, physical therapists, and other paramedics if needed. | *Staffing:* An interdisciplinary team of healthcare professionals with geriatric expertise, including a geriatrician and/or medical specialist(s), elderly care physician, nurses, physical therapists, and other paramedics if needed. |
| *Coordinating practitioner:* The elderly care physician, nurse practitioner, or physician assistant. | *Coordinating practitioner:* The elderly care physician (high-complex STRC), general practitioner (low-complex STRC), nurse practitioner, or physician assistant. | *Coordinating practitioner:* The geriatrician, elderly care physician, nurse practitioner, or physician assistant. |
| *Treatment:*  A multidisciplinary set of evaluative, diagnostic and therapeutic interventions that are adapted to the rehabilitation needs of the frail elderly individual.  Palliative care cannot be provided in GR settings. | *Treatment:*  Three different STRC care paths exist:  - STRC low complex provides regular care for patients who are not in need for specific paramedic treatment, but temporarily need more care than homecare can provide.  - STRC high complex provides not only increased care, but also (multidisciplinary) treatment or rehabilitation in a slower pace than geriatric revalidation.  - STRC palliative care provides care for patients in the last 3 months of their life. | *Treatment:*  The four AGCH care components are:  (1) low-complex acute specialized geriatric care is safely provided  (2) care is patient-centred and focused on rehabilitation and return home (e.g. CGA, ACP, early rehabilitation, function focused care, caregivers involved during treatment (decisions))  (3) integrated care: transmural and close to home (e.g. comprehensive discharge planning (caregiver involved), warm handover to the GP and district nurse)  (4) fitting environment to prevent delirium and functional decline (e.g. rooming-in, noise reduction, management of delirium-inducing drugs).  Palliative care can be provided in AGCH settings. |
| *Funding:* Treatment, therapy and ADL care is funded according to a Diagnosis Treatment Combination (DTC).  --> For patients with a LTC indication, a LTC financing label ZZP 9b can be used. | *Funding:* Treatment, therapy and ADL care is funded according to a daily tariff, for a maximum of 6 months:  - STRC low complex: GP and paramedic treatment payment according to regular tariffs (as if patient would be home).  - STRC high complex: up to 1,5 hour treatment per week by ECP/paramedics.  - STRC hospice care: up to 3 hours treatment per week by GP/ECP/paramedics is funded.  --> For patients with a LTC indication, the LTC crisis financing labels (somatic or psychogeriatric) can be used. | *Funding:* Treatment, therapy and ADL care is funded according to a daily tariff, which is currently (2023) provided through an experimental financing structure.  --> For patients with a LTC indication no separate financing label has to be used under the experimental financing structure. |

*Abbreviations: ACP = Advance Care Planning; AGCH = Acute Geriatric Community Hospital; CGA = comprehensive geriatric assessment, COPD = chronic obstructive pulmonary disorder; CT = computed tomography; ED = emergency department; LTC = long-term care, MRI = magnetic resonance imaging; STRC = Short-Term Residential Care.*

Appendix 2: Checklist for reporting (STROBE & RECORD)

**The RECORD statement – checklist of items, extended from the STROBE statement, that should be reported in observational studies using routinely collected health data.**

|  | **Item No.** | **STROBE items** | **Location in the manuscript where items are reported** | **RECORD items** | **Location in the manuscript where items are reported** |
| --- | --- | --- | --- | --- | --- |
| **Title and abstract** | | | | | |
|  | 1 | (a) Indicate the study’s design with a commonly used term in the title or the abstract (b) Provide in the abstract an informative and balanced summary of what was done and what was found | Page 1  Page 1 | RECORD 1.1: The type of data used should be specified in the title or abstract. When possible, the name of the databases used should be included.  RECORD 1.2: If applicable, the geographic region and timeframe within which the study took place should be reported in the title or abstract.  RECORD 1.3: If linkage between databases was conducted for the study, this should be clearly stated in the title or abstract. | Page 1  Page 1  Page 1 |
| **Introduction** | | | | | |
| Background rationale | 2 | Explain the scientific background and rationale for the investigation being reported | Page 2 |  |  |
| Objectives | 3 | State specific objectives, including any prespecified hypotheses | Page 2-3 |  |  |
| **Methods** | | | | | |
| Study Design | 4 | Present key elements of study design early in the paper | Page 3 |  |  |
| Setting | 5 | Describe the setting, locations, and relevant dates, including periods of recruitment, exposure, follow-up, and data collection | Page 3 |  |  |
| Participants | 6 | *(a) Cohort study* - Give the eligibility criteria, and the sources and methods of selection of participants. Describe methods of follow-up  *Case-control study* - Give the eligibility criteria, and the sources and methods of case ascertainment and control selection. Give the rationale for the choice of cases and controls  *Cross-sectional study* - Give the eligibility criteria, and the sources and methods of selection of participants  *(b) Cohort study* - For matched studies, give matching criteria and number of exposed and unexposed  *Case-control study* - For matched studies, give matching criteria and the number of controls per case | Page 3  N/A | RECORD 6.1: The methods of study population selection (such as codes or algorithms used to identify subjects) should be listed in detail. If this is not possible, an explanation should be provided.  RECORD 6.2: Any validation studies of the codes or algorithms used to select the population should be referenced. If validation was conducted for this study and not published elsewhere, detailed methods and results should be provided.  RECORD 6.3: If the study involved linkage of databases, consider use of a flow diagram or other graphical display to demonstrate the data linkage process, including the number of individuals with linked data at each stage. | Pages 3 and 4  Appendix 4  Page 3  Appendix 3 |
| Variables | 7 | Clearly define all outcomes, exposures, predictors, potential confounders, and effect modifiers. Give diagnostic criteria, if applicable. | Page 4, Appendix 3 | RECORD 7.1: A complete list of codes and algorithms used to classify exposures, outcomes, confounders, and effect modifiers should be provided. If these cannot be reported, an explanation should be provided. | Appendix 3 |
| Data sources/ measurement | 8 | For each variable of interest, give sources of data and details of methods of assessment (measurement).  Describe comparability of assessment methods if there is more than one group | Appendix 3 |  |  |
| Bias | 9 | Describe any efforts to address potential sources of bias | Page 3 |  |  |
| Study size | 10 | Explain how the study size was arrived at | Appendix 4 |  |  |
| Quantitative variables | 11 | Explain how quantitative variables were handled in the analyses. If applicable, describe which groupings were chosen, and why | Page 4, Appendix 1, 3 |  |  |
| Statistical methods | 12 | (a) Describe all statistical methods, including those used to control for confounding  (b) Describe any methods used to examine subgroups and interactions  (c) Explain how missing data were addressed  (d) *Cohort study* - If applicable, explain how loss to follow-up was addressed  *Case-control study* - If applicable, explain how matching of cases and controls was addressed  *Cross-sectional study* - If applicable, describe analytical methods taking account of sampling strategy  (e) Describe any sensitivity analyses | Page 3-4  Page 3-4, and Appendix 5  Appendix 3  N/A  N/A |  |  |
| Data access and cleaning methods |  | .. |  | RECORD 12.1: Authors should describe the extent to which the investigators had access to the database population used to create the study population.  RECORD 12.2: Authors should provide information on the data cleaning methods used in the study. | Page 3  Page 3  Appendix 3 |
| Linkage |  | .. |  | RECORD 12.3: State whether the study included person-level, institutional-level, or other data linkage across two or more databases. The methods of linkage and methods of linkage quality evaluation should be provided. | Page 3 |
| **Results** | | | | | |
| Participants | 13 | (a) Report the numbers of individuals at each stage of the study (*e.g.*, numbers potentially eligible, examined for eligibility, confirmed eligible, included in the study, completing follow-up, and analysed)  (b) Give reasons for non-participation at each stage.  (c) Consider use of a flow diagram | Appendix 4  N/A  Appendix 4 | RECORD 13.1: Describe in detail the selection of the persons included in the study (*i.e.,* study population selection) including filtering based on data quality, data availability and linkage. The selection of included persons can be described in the text and/or by means of the study flow diagram. | Page 3 and Appendix 4 |
| Descriptive data | 14 | (a) Give characteristics of study participants (*e.g.*, demographic, clinical, social) and information on exposures and potential confounders  (b) Indicate the number of participants with missing data for each variable of interest  (c) *Cohort study* - summarise follow-up time (*e.g.*, average and total amount) | Page 4 and 5, Tables 1 and 2, and Appendix 7  N/A  Page 5, Tables 1 and 2, and Appendix 6 |  |  |
| Outcome data | 15 | *Cohort study* - Report numbers of outcome events or summary measures over time  *Case-control study* - Report numbers in each exposure category, or summary measures of exposure  *Cross-sectional study* - Report numbers of outcome events or summary measures | Pages 5 and 6, Figures 1,2 and 3, Appendix 6 |  |  |
| Main results | 16 | (a) Give unadjusted estimates and, if applicable, confounder-adjusted estimates and their precision (e.g., 95% confidence interval). Make clear which confounders were adjusted for and why they were included  (b) Report category boundaries when continuous variables were categorized  (c) If relevant, consider translating estimates of relative risk into absolute risk for a meaningful time period | Page 6  Figure 3  Appendix 5 |  |  |
| Other analyses | 17 | Report other analyses done—e.g., analyses of subgroups and interactions, and sensitivity analyses | Page 5-6, Appendix 7 |  |  |
| **Discussion** | | | | | |
| Key results | 18 | Summarise key results with reference to study objectives | Page 6-7 |  |  |
| Limitations | 19 | Discuss limitations of the study, taking into account sources of potential bias or imprecision. Discuss both direction and magnitude of any potential bias | Page 7 | RECORD 19.1: Discuss the implications of using data that were not created or collected to answer the specific research question(s). Include discussion of misclassification bias, unmeasured confounding, missing data, and changing eligibility over time, as they pertain to the study being reported. | Page 8 |
| Interpretation | 20 | Give a cautious overall interpretation of results considering objectives, limitations, multiplicity of analyses, results from similar studies, and other relevant evidence | Page 7-8 |  |  |
| Generalisability | 21 | Discuss the generalisability (external validity) of the study results | Page 8 |  |  |
| **Other Information** | | | | | |
| Funding | 22 | Give the source of funding and the role of the funders for the present study and, if applicable, for the original study on which the present article is based | Title page |  |  |
| Accessibility of protocol, raw data, and programming code |  | .. |  | RECORD 22.1: Authors should provide information on how to access any supplemental information such as the study protocol, raw data, or programming code. | Title page |

Appendix 3: Variables with data sources and underlying assumptions

| **Variable** | **Dataset and version used** | **Details and underlying assumptions** |
| --- | --- | --- |
| Geriatric rehabilitation costs  Hospital admission costs  Outpatient hospital care costs | MSZPrestatiesVEKT2022TABV2  MSZZorgactiviteitenVEKT2022TABV2 | Based on the claimed costs for medical specialist care in 2022. Costs were equally distributed across the days between the first and last claimed activity.  For hospital care, a distinction was made between hospital admission costs (defined as costs claims on days with inpatient hospitalisation and/or visits to the emergency department) and outpatient costs (i.e. costs claims on days without inpatient hospitalisation and/or visits to the emergency department). |
| District care costs | ZVWWVP2022TABV2 | Based on the claimed cost for district nursing care use and costs in 2022. Costs were equally distributed across the first and last dates of the claim. |
| Nursing home long-term care costs  Long-term care at home costs | WLZZIN2022TABV3 | Based on the claimed costs for long-term care in 2022. Costs were equally distributed across the first and last dates of the claim. Groups were formed according to the different levels of care intensity (zorgzwaartepakketten).These groups were subsequently categorised into institutional long-term care (called nursing home long-term care) and non-institutional long-term care (long-term care at home). |
| STRC costs | ZVWELV2022TABV1 | Based on the claimed costs for STRC admission in 2022. Costs were equally distributed across the first and last dates of STRC admission. |
| Sex | GBAPERSOON2022TABV2 | Registered sex. |
| Age | GBAPERSOON2022TABV2 | Age was calculated based on the date of birth and the date of admission to GR, ensuring that age reflects the moment of admission. |
| Migration background | GBAPERSOON2022TABV2 | A dummy describing whether the individual has a first-generation migration background (i.e., the individual was not born in the Netherlands). |
| Living situation | GBAHUISHOUDENS2022BUSV1 | The dataset includes information on an individual's living situation throughout time (longitudinal). Information on household composition on the day before GR admission was used. The categories ‘living with or without children, living alone with or without children, residing in an institution, or unknown’ were recategorized into four groups: living alone, living with others, institutionalised, and unknown. |
| Income | INHA2021TABV1 | Registered household income in 2021 was compared to the social minimum income of that year. Low income was defined as up to 140% of the social minimum, middle income as 140%–200%, and high income as above 200% of the social minimum. |
| Medication use | MEDICIJNTAB2021TABV1 | Medication dispensed by primary care pharmacies in 2021, based on ATC4 codes (thus excluding medication provided by hospitals and under the LTC-act). Each unique ATC4 code was considered a distinct type of medication used by the patient. |
| Psychotropic drug use | MEDICIJNTAB2021TABV1 | A dummy variable equal to one if the individual was dispensed one or more medications in 2021 with the following ATC4 codes: N05A (antipsychotics), N05B (anxiolytics), N05CD (benzodiazepines), N06A (antidepressants), or N06C (antidepressant combinations). |
| Dementia | DEMENTIEPOPULATIEVEKTIS2022TABV1 | A dummy variable equal to one if Vektis determined, based on their revalidated method, that the individual had dementia in 2022 or earlier. See <https://www.vektis.nl/herijking-methode-bepaling-dementiepopulatie> |
| Primary diagnosis | MSZPrestatiesVEKT2022TABV2  MSZZorgactiviteitenVEKT2022TABV2 | The declared diagnosis codes for GR. The same classification as Vektis was applied: [https://www.vektis.nl/intelligence/publicaties/factsheet-geriatrische-revalidatiezorg](https://www.vektis.nl/intelligence/publicaties/factsheet-geriatrische-revalidatiezorg) |
| Length of stay GR | MSZPrestatiesVEKT2022TABV2  MSZZorgactiviteitenVEKT2022TABV2 | Registered nursing days were summed when consecutive, with the assumption that an interval of more than two days between stays indicates a readmission. |
| Length of home-based GR | MSZPrestatiesVEKT2022TABV2  MSZZorgactiviteitenVEKT2022TABV2 | Home-based GR was defined as cases in which one or more treatment components were registered on a day within a GR DBC, but no nursing day was recorded on that day. The duration of home-based GR was calculated as the time between the first and last registered home-based GR treatment component within a Diagnosis Treatment Combination (DTC). |
| Readmission (GR, ED, hospital) | MSZPrestatiesVEKT2022TABV2  MSZZorgactiviteitenVEKT2022TABV2 | Dummy variables set to one if, between GR discharge and the end of the six-month trajectory, at least one GR nursing day, ED visit, or hospital nursing day was claimed. |
| Inflow  Outflow | GEBWMOTABV12022  ZVWWVP2022TABV2  WLZZIN2022TABV3  ZVWELV2022TABV1  MSZPrestatiesVEKT2022TABV2  MSZZorgactiviteitenVEKT2022TABV2 | Categorical variables describing the highest care use in the two weeks before GR admission (inflow) and the two weeks after GR admission (outflow). The ranking of care use was as follows (from low to high): home without formal care (no care use), home with household help (social care use), home with district care, LTC at home, nursing home care, STRC admission, GR admission, hospital admission, ED admission, ED & hospital admission. |
| Survival days | GBAOVERLIJDEN2022TABV1 | The number of days a person was alive during the six-month period (181.5 days) was calculated as the time between the start date and either the date of death (if occurring within the six-month trajectory) or the end of the six months. |
| Costs per survival day | GBAOVERLIJDEN2022TABV1 | Costs per survival day were calculated by dividing the total mean costs by the number of survival days. |
| Data cleaning procedures |  | We conducted several data cleaning steps before analysis. Implausible values were identified and addressed for variables such as age, healthcare utilisation, costs, and mortality. Duplicate patient identities were removed. Diagnoses were checked for internal consistency and plausibility. We also examined the dataset for duplicate cost entries. Missing data were labelled as ‘unknown’ and reported transparently in the descriptive tables. |
| Data Security Guidelines for Claims Data Processing |  | To prevent disclosure of participant identity, the following guidelines were applied:  1. Minimum number of observations: All outputs include at least 10 units (unweighted) as the basis for each cell or data point.  2. Models: All modelled outputs must have a minimum of 10 degrees of freedom, where degrees of freedom are calculated as the number of observations minus the number of parameters and other model constraints. Residuals and residual plots are not disclosed.  3. Group disclosure: In all tables and similar outputs, no single cell may contain more than 90% of the total units in the respective row or column to prevent group disclosure.  4. Dominance: In all tables and similar data, the largest contributor to a cell must not account for more than 50% of the cell total. |

# Appendix 4: Consort diagram

**Fig. A4.1.** Consort diagram


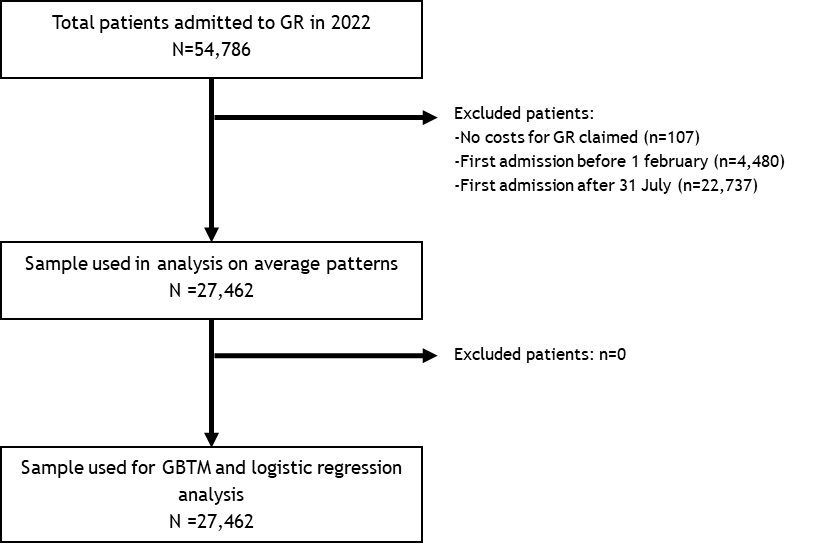


# Appendix 5: Group-based trajectory modelling specifications

**Table A5.1.** Correlation matrix total trajectory costs over time (TC_1 – TC_6) for group-based trajectory modelling (GBTM) cohort (n = 27,462).

|  | **TC_1** | **TC_2** | **TC_3** | **TC_4** | **TC_5** | **TC_6** |
| --- | --- | --- | --- | --- | --- | --- |
| **TC_1** | 1.0000 |  |  |  |  |  |
| **TC_2** | 0.9332 | 1.0000 |  |  |  |  |
| **TC_3** | 0.8160 | 0.9284 | 1.0000 |  |  |  |
| **TC_4** | 0.7142 | 0.8340 | 0.9607 | 1.0000 |  |  |
| **TC_5** | 0.6367 | 0.7565 | 0.9044 | 0.9779 | 1.0000 |  |
| **TC_6** | 0.5725 | 0.6901 | 0.8490 | 0.9408 | 0.9849 | 1.0000 |

**Table A5.2.** Bayesian Information Criteria, Akaike Information Criterion and group membership <5% of crude trajectory calculations with fixed quadratic growth terms used to select the adequate number of groups for the GBTM cohort (n = 27,462).

| **Number of groups** | **BIC (n = 13,001)** | **AIC** | **Group membership <5%** |
| --- | --- | --- | --- |
| 1 | -909,612.14 | -909,595.70 | - |
| 2 | -893,461.50 | -893,428.61 | 0 |
| 3 | -883,163.94 | -883,114.62 | 1 |
| 4 | -881,479.53 | -881,413.76 | 2 |
| 5 | -900,991.37 | -900,909.16 | 3 |
| 6 | -893,543.26 | -893,444.61 | 4 |

**Table A5.3.** Bayesian Information Criteria, Akaike Information Criterion and group membership <5% of crude trajectory calculations with two groups used to select the adequate growth terms for the GBTM cohort (n = 27,462).

| **Growth terms** | **BIC (n = 13,001)** | **AIC** | **Group membership <5%** |
| --- | --- | --- | --- |
| Linear, linear | -895,364.43 | -895,339.77 | 0 |
| Linear, quadratic | -895,129.26 | -895,100.49 | 0 |
| Quadratic, linear | -893,698.86 | -893,670.09 | 0 |
| Quadratic, quadratic | -893,461.50 | -893,428.61 | 0 |
| Quadratic, cubic | -893,466.61 | -893,429.61 | 0 |
| Cubic, quadratic | -893,466.61 | -893,429.61 | 0 |
| Cubic, cubic | -910,995.95 | -910,954.85 | 0 |

**Figure A5.4.** Graph of GBTM results: two cubic groups with confidence intervals (n = 27,462).


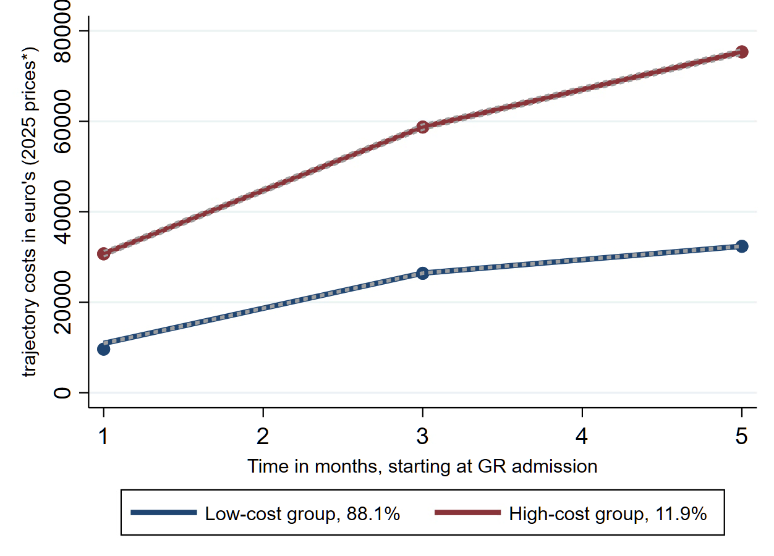


**Table A5.5.** Posterior diagnostics of model performance of basic trajectory model (n = 27,462)

| **Group** | **Model estimate (π^)** | **Proportion classified (p^)** | **Ave. posterior probability** | **Odds correct classification** |
| --- | --- | --- | --- | --- |
| 1 (Higher-cost) | 0.116 | 0.119 | 0.93 | 100.88 |
| 2 (Lower-cost) | 0.884 | 0.881 | 0.99 | 10.10 |

# Appendix 6: Results

**Table A6.1.** Average longitudinal pattern in trajectory costs and healthcare utilisation for the full GR cohort (n = 27,462). GR = geriatric rehabilitation, LTC = long-term care, STRC = short-term residential care

|  | **Month 1** | **Month 2** | **Month 3** | **Month 4** | **Month 5** | **Month 6** | **Total (% of TTC)** |
| --- | --- | --- | --- | --- | --- | --- | --- |
| **GR costs (% of total monthly costs)** | €12(0%) | €9,648 (79%) | €3,523(60%) | €1,299 (32%) | €525 (16%) | €275 (9%) | **€15,282 (38%)** |
| **GR use (% of patients)** | 0% | 100% | 54% | 21% | 9% | 4% | **100%** |
|  |  |  |  |  |  |  |  |
| **District care costs (% of total monthly costs)** | €301 (2%) | €171 (1%) | €450 (8%) | €496 (12%) | €438 (14%) | €412 (14%) | **€2,266 (6%)** |
| **District care use (% of patients)** | 35% | 30% | 43% | 41% | 37% | 34% | **66%** |
|  |  |  |  |  |  |  |  |
| **LTC at home costs (% of total monthly costs)** | €62 (1%) | €33 (0%) | €65 (1%) | €107 (3%) | €133 (4%) | €156 (5%) | **€556 (1%)** |
| **LTC at home use (% of patients)** | 3% | 2% | 3% | 4% | 5% | 5% | **7%** |
|  |  |  |  |  |  |  |  |
| **Nursing home LTC costs (% of total monthly costs)** | €57 (0%) | €98 (1%) | €453 (8%) | €948 (24%) | €1,157 (36%) | €1,280 (43%) | **€3,994 (10%)** |
| **Nursing home LTC use (% of patients)** | 1% | 2% | 8% | 13% | 14% | 15% | **17%** |
|  |  |  |  |  |  |  |  |
| **STRC costs (% of total monthly costs)** | €199 (2%) | €47 (0%) | €98 (2%) | €107 (3%) | €87 (3%) | €70 (2%) | **€608 (2%)** |
| **STRC use (% of patients)** | 4% | 2% | 2% | 2% | 2% | 1% | **9%** |
|  |  |  |  |  |  |  |  |
| **Hospital admission costs (% of total monthly costs)** | €6,773 (56%) | €1.081 (9%) | € 547 (9%) | €473 (12%) | €385 (12%) | €338 (11%) | **€9,597 (24%)** |
| **Hospital admission use (% of patients)** | 90% | 55% | 12% | 10% | 8% | 7% | **94%** |
|  |  |  |  |  |  |  |  |
| **Outpatient care costs (% of total monthly costs)** | €4,743 (39%) | €1,123 (9%) | €762 (13%) | €588 (15%) | €500 (15%) | €449 (15%) | **€8,164 (20%)** |
| **Outpatient care use (% of patients)** | 89% | 77% | 67% | 59% | 52% | 49% | **99%** |
|  |  |  |  |  |  |  |  |
| **Total costs per category (% of TTC)** | **€12,147 (30%)** | **€12,202 (30%)** | **€5,898(15%)** | **€4,017 (10%)** | **€3,225 (9%)** | **€2,980 (7%)** | **40,469€ (100%)** |

**Table A6.2.** Longitudinal average pattern in trajectory costs and healthcare utilisation for the high-cost group (n = 3,182). GR = geriatric rehabilitation, LTC = long-term care, STRC = short-term residential care

|  | **Month 1** | **Month 2** | **Month 3** | **Month 4** | **Month 5** | **Month 6** | **Total (% of TTC)** |
| --- | --- | --- | --- | --- | --- | --- | --- |
| **GR costs (% of total monthly costs)** | €25 (0%) | €10,735 (66%) | €6,036 (51%) | €3,284 (35%) | €1,548 (21%) | €742 (13%) | **€22,369 (27%)** |
| **GR use (% of patients)** | 1% | 100% | 73% | 45% | 24% | 11% | **100%** |
|  |  |  |  |  |  |  |  |
| **District care costs (% of total monthly costs)** | €211 (1%) | €102 (1%) | €388 (3%) | €556 (6%) | €499 (7%) | €443(7%) | **€2,199 (3%)** |
| **District care use (% of patients)** | 26% | 15% | 26% | 31% | 30% | 29% | **56%** |
|  |  |  |  |  |  |  |  |
| **LTC at home costs (% of total monthly costs)** | €116 (0%) | €92 (1%) | €87 (1%) | €104 (1%) | €128 (2%) | €173 (3%) | **€700 (1%)** |
| **LTC at home use (% of patients)** | 3% | 2% | 2% | 3% | 4% | 4% | **7%** |
|  |  |  |  |  |  |  |  |
| **Nursing home LTC costs (% of total monthly costs)** | €249 (1%) | €289 (2%) | €750 (6%) | €1,576 (17%) | €2,052 (28%) | €2,291 (39%) | **€7,207 (9%)** |
| **Nursing home LTC use (% of patients)** | 4% | 5% | 12% | 20% | 25% | 26% | **29%** |
|  |  |  |  |  |  |  |  |
| **STRC costs (% of total monthly costs)** | €111 (0%) | €51 (0%) | €190 (2%) | €230 (2%) | €202 (3%) | €152 (3%) | **€936 (1%)** |
| **STRC use (% of patients)** | 3% | 2% | 4% | 4% | 3% | 3% | **10%** |
|  |  |  |  |  |  |  |  |
| **Hospital admission costs (% of total monthly costs)** | €12,268 (39%) | €2,057 (13%) | €2,046 (17%) | €1,769 (19%) | €1,316 (18%) | €911 ( 15%) | **€20,368(25%)** |
| **Hospital admission use (% of patients)** | 96% | 66% | 30% | 23% | 18% | 13% | **98%** |
|  |  |  |  |  |  |  |  |
| **Outpatient care costs (% of total monthly costs)** | €18,308 (59%) | €3,000 (18%) | €2,342 (20%) | €1,874 (20%) | €1,500 (21%) | €1,210 (20%) | **€28,234 (34%)** |
| **Outpatient care use (% of patients)** | 99 % | 89% | 79% | 72% | 64% | 59% | **100%** |
|  |  |  |  |  |  |  |  |
| **Total costs per category (% of TTC)** | **€31,287 (38%)** | **€16,326 (20%)** | **€11,838 (14%)** | **€9,394 (11%)** | **€7,245 (9%)** | **€5,922 (7%)** | **€82,013 (100%)** |

**Table A6.3.** Longitudinal average pattern in trajectory costs and healthcare utilisation for the low-cost group (n = 24,280). GR = geriatric rehabilitation, LTC = long-term care, STRC = short-term residential care

|  | **Month 1** | **Month 2** | **Month 3** | **Month 4** | **Month 5** | **Month 6** | **Total (% of TTC)** |
| --- | --- | --- | --- | --- | --- | --- | --- |
| **GR costs (% of total monthly costs)** | €10 (0%) | €9,506 (82%) | €3,194 (62%) | €1.039 (31%) | €391 (14%) | €213 (8%) | **€14,375(41%)** |
| **GR use (% of patients)** | 0% | 100% | 52% | 18% | 7% | 3% | **100%** |
|  |  |  |  |  |  |  |  |
| **District care costs (% of total monthly costs)** | €312 (3%) | €180 (2%) | €458 (9%) | €488 (15%) | €430 (16%) | €408 (16%) | **€2,263 (6%)** |
| **District care use (% of patients)** | 36% | 31% | 44% | 42% | 38% | 35% | **67%** |
|  |  |  |  |  |  |  |  |
| **LTC at home costs (% of total monthly costs)** | €55 (1%) | €26 (0%) | €60 (1%) | €108 (3%) | €134 (5%) | €153 (6%) | **€534 (2%)** |
| **LTC at home use (% of patients)** | 3% | 2% | 3% | 4% | 5% | 5% | **7%** |
|  |  |  |  |  |  |  |  |
| **Nursing home LTC costs (% of total monthly costs)** | €32 ( 0%) | €73 (1%) | €415 (8%) | €866 (26%) | €1,039 (39%) | €1,148 (44%) | **€3,557 (10%)** |
| **Nursing home LTC use (% of patients)** | 1% | 2% | 8% | 12% | 13% | 13% | **16%** |
|  |  |  |  |  |  |  |  |
| **STRC costs (% of total monthly costs)** | €210 (2%) | €47 (0%) | €86 (2%) | €90 (3%) | €72 (3%) | €60 (2%) | **€563 (2%)** |
| **STRC use (% of patients)** | 5% | 2% | 2% | 2% | 1% | 1% | **9%** |
|  |  |  |  |  |  |  |  |
| **Hospital admission costs (% of total monthly costs)** | €6,053 (63%) | €953 (8%) | €350 (7%) | €303 (9%) | €263 (10%) | €263 (10%) | **€8,184 (23%)** |
| **Hospital admission use (% of patients)** | 89% | 54% | 9% | 8% | 7% | 6% | **93%** |
|  |  |  |  |  |  |  |  |
| **Outpatients care costs (% of total monthly costs)** | €2,965 (31%) | €878 (8%) | €555 (11%) | €419 (13%) | €369 (14%) | €349 (13%) | **€5,525 (16%)** |
| **Outpatient care use (% of patients)** | 88% | 75% | 66% | 57% | 50% | 47% | **99%** |
|  |  |  |  |  |  |  |  |
| **Total costs per category (% of TTC)** | **€9,639 (28%)** | **€11,661 (33%)** | **€5,119(15%)** | **€3.313 (9%)** | **€2,698 (8%)** | **€2,594 (7%)** | **€35,024 (100%)** |

**Table A6.4.** Results of logistic linear model for membership to the high- (versus low-) cost group (n =27,462 )

| **Higher vs. lower-cost group** | **Odds Ratio (SD)** | **95% CI** | **P>\|z\|** | **Lower cost-group** |
| --- | --- | --- | --- | --- |
| Male | 1.36 (0.06) | 1.25 - 1.47 | 0.000 | *Reference* |
| Age | 0.96 (0.00) | 0.95 - 0.96 | 0.000 | *Reference* |
| Living situation |  |  |  |  |
| Living alone | *Reference* | *Reference* | *Reference* | *Reference* |
| Living together | 1.24 (0.05) | 1.14 – 1.34 | 0.000 | *Reference* |
| Living in an institution | 4.51 (0.50) | 3.63 – 5.62 | 0.000 | *Reference* |
| Unknown | 1.40 (0.39) | 0.82 – 2.40 | 0.213 | *Reference* |
| Medicine count 2021 | 1.01 (0.00) | 0.99 – 1.01 | 0.103 | *Reference* |
| Dementia | 1.00 (0.06) | 0.89 – 1.13 | 0.996 | *Reference* |
|  |  |  |  |  |
| Primary diagnosis |  |  |  |  |
| Stroke | 1.85 (0.12) | 1.62 – 2.10 | 0.000 | *Reference* |
| Trauma | *Reference** | *Reference* | *Reference* | *Reference* |
| Elective surgery | 0.41 (0.05) | 0.32 – 0.51 | 0.000 | *Reference* |
| Amputation | 4.11 (0.42) | 3.38 – 5.02 | 0.000 | *Reference* |
| Oncological condition | 4.03 (0.35) | 3.39 – 4.78 | 0.000 | *Reference* |
| Cardiovascular condition | 4.70 (0.41) | 3.96 – 5.58 | 0.000 | *Reference* |
| Respiratory condition | 1.90 (0.14) | 1.65 – 2.19 | 0.000 | *Reference* |
| Organ failure | 2.42 (0.17) | 2.12 – 2.77 | 0.000 | *Reference* |
| Other | 1.71 (0.13) | 1.48 – 1.99 | 0.000 | *Reference* |
|  |  |  |  |  |
| _cons | 1.83(0.29) | 1.34 – 2.50 | 0.000 | *Reference* |
| Sample-n | 8,490 |  |  | 27,462 |

**Trauma was selected as the reference category because it represents the largest diagnostic group (n = 8,038), has an average length of geriatric rehabilitation stay, and serves as a broad and clinically distinct category that contrasts well with other diagnostic groups.*

# Appendix 7: Home-based geriatric rehabilitation

**Table A7.1.** Patient and trajectory characteristics of the home-based geriatric rehabilitation group (n= 3,067)

| **Home-based GR**  **Patient characteristics** | **A) Total cohort**  **N = 3,067** | **B) High-cost group**  **N = 505** | **C) Low-cost group**  **N = 2,562** |
| --- | --- | --- | --- |
| Male, N(%) | 1,428 (46.6%) | 295 (58.4%) | 1,133 (44.2%) |
| Age, mean (SD) | 77 (9.8) | 73 (10.1) | 78 (9.6) |
| Migration background | 314 ( 10.2%) | 68 ( 13.5%) | 246 (9.6%) |
| Living situation before GR admission, N(%) |  |  |  |
| Living alone | 1,574 (51.3%) | 210 (41.6%) | 1,364(53.2%) |
| Living together | 1,441 (47.0%) | 280 (55.5%) | 1,161 (45.3%) |
| Living in institution | 41 ( 1.3%) | 13 ( 2.6%) | 28 ( 1.1%) |
| Missing | 11 ( 0.4%) | 2 ( 0.4%) | 9 ( 0.4%) |
| Income of household in 2021, N(%)* |  |  |  |
| Low income | 915 (29.8%) | 131 (25.9%) | 784 (30.6%) |
| Middle income | 763 (24.9%) | 120 (23.8%) | 643 (25.1%) |
| High income | 1,350 (44.0%) | 240 (47.5%) | 1,110(43.3%) |
| Missing | 39 ( 1.3%) | 14 ( 2.8%) | 25 ( 1.0%) |
| Medication use in 2021, N(%)** |  |  |  |
| 0 | 120 ( 3.9%) | 30 ( 5.9%) | 90 ( 3.5%) |
| 1-4 | 579 (18.9%) | 86 (17.0%) | 493 ( 19.2%) |
| 5-9 | 1,123 (36.6%) | 158 (31.3%) | 965 (37.7%) |
| 10-14 | 823 (26.8%) | 138 (27.3%) | 685(26.7%) |
| 15-19 | 311 (10.1%) | 60 (11.9%) | 251 (9.8%) |
| >20 | 111 ( 3.6%) | 33 ( 6.5%) | 78 ( 3.0%) |
| n≥1 psychotropic drugs use in 2021, N(%)^†^ | 613 (20.0%) | 88 (17.4%) | 525 (20.5%) |
| Dementia^‡^ | 360 (11.7%) | 42 (8.3%) | 318 (12.4%) |
| **Primary diagnosis: GR admission** | **A) Total cohort**  **N = 3,067** | **B) High-cost group**  **N = 505** | **C) Low-cost group**  **N = 2,562** |
| Stroke | 717 (23.4%) | 103 (20.4%) | 614 (24.0%) |
| Trauma | 743 (24.2%) | 84 (16.6%) | 659 (25.7%) |
| Elective surgery | 263 ( 8.6%) | 13 ( 2.6%) | 250 ( 9.8%) |
| Amputation | 114 ( 3.7%) | 37 ( 7.3%) | 77 ( 3.0%) |
| Oncological condition | 128 ( 4.2%) | 40 ( 7.9%) | 88 ( 3.4%) |
| Cardiovascular condition | 118 ( 3.9%) | 38 ( 7.5%) | 80 ( 3.1%) |
| Respiratory condition | 330 (10.8%) | 57 (11.3%) | 273 (10.7%) |
| Organ failure | 326 (10.6%) | 81 (16.0%) | 245 ( 9.6%) |
| Other | 328 (10.7%) | 52 (10.3%) | 276 (10.8%) |
| **Trajectory characteristics** | **A) Total cohort**  **N = 3,067** | **B) High-cost group**  **N = 505** | **C) Low-cost group**  **N = 2,562** |
| Trajectory costs |  |  |  |
| Mean trajectory costs (SD) | €44,869 (€24,926) | €83,860 (€24,948) | €37,183 (€16,200) |
| Median trajectory costs | €39,933 | €79,187 | €34,620 |
| LOS first admission GR |  |  |  |
| Mean LOS (SD) | 33.4 (26.3) | 38.7 (31.4) | 32.3 (25.0) |
| Median LOS | 28 | 31 | 27 |
| LOS first admission home-based GR |  |  |  |
| Mean LOS (SD) | 13.5(21.5) | 9.9(17.6) | 14.2(22.1) |
| Median LOS | 3 | 2 | 3 |
| Readmissions (after GR discharge) |  |  |  |
| ≥1 GR readmission(s) | 833 (27.2%) | 265 (52.5%) | 568 (22.2%) |
| ≥1 ED readmission(s) | 825 (26.9%) | 204 (40.4%) | 621 (24.2%) |
| ≥1 hospital admission day(s) | 1,098 (35.8%) | 330 (65.4%) | 768 (30.0%) |
| Survival |  |  |  |
| Death during 6 6-month trajectory | 332 (10.8%) | 76 (15.1%) | 256 (10.0%) |
| Survival days, mean (SD) | 173 (29.6) | 172 (28.5) | 173 (29.8) |
| Costs per survival day (SD) | €273 (€176) | €510 (€223) | €226 (€117) |

*SD = standard deviation, GR = geriatric rehabilitation, ED = emergency department, LTC = long-term care, LOS = length of stay, STRC = short-term residential care. * Low income is up to 140% of social minimum income, middle income is 140%-200%, and high income is more than 200% of social minimum income. ** Based on ATC-code system, does not include medication provided in hospitals or under the LTC act in nursing homes. † Based on Anatomic Therapeutic Chemical Codes N05A(antipsychotics), N05B(anxiolytics), N05CD(benzodiazepine), N06A(antidepressants) and N06C (antidepressants in combination with psycholeptics). ‡* *Survival days calculated over a 6-month(182.5 days) follow-up period*
